# Genomic and phenotypic characterization of *Acinetobacter colistiniresistens* isolated from the feces of a healthy member of the community

**DOI:** 10.1038/s41598-023-39642-0

**Published:** 2023-08-03

**Authors:** Nazmul Hasan Muzahid, Muhammad Zarul Hanifah Md Zoqratt, Kah Ern Ten, Md Hamed Hussain, Tin Tin Su, Qasim Ayub, Hock Siew Tan, Sadequr Rahman

**Affiliations:** 1 School of Science, Monash University Malaysia, Bandar Sunway, 47500 Subang Jaya, Selangor Darul Ehsan Malaysia; 2 South East Asia Community Observatory (SEACO), Global Public Health, Jeffrey Cheah School of Medicine and Health Sciences, Monash University Malaysia, Bandar Sunway, 47500 Subang Jaya, Selangor Malaysia; 3 Monash University Malaysia Genomics Facility, Bandar Sunway, 47500 Subang Jaya, Selangor Darul Ehsan Malaysia; 4 Tropical Medicine & Biology Multidisciplinary Platform, Monash University Malaysia, Bandar Sunway, 47500 Subang Jaya, Selangor Malaysia

**Keywords:** Bacteriology, Bacterial pathogenesis, Bacterial genomics

## Abstract

*Acinetobacter* species are widely known opportunistic pathogens causing severe community and healthcare-associated infections. One such emerging pathogen, *Acinetobacter colistiniresistens,* is known to exhibit intrinsic resistance to colistin. We investigated the molecular characteristics of *A. colistiniresistens* strain C-214, isolated from the fecal sample of a healthy community member, as part of a cohort study being conducted in Segamat, Malaysia. Comparison of the whole genome sequence of C-214 with other *A. colistiniresistens* sequences retrieved from the NCBI database showed 95% sequence identity or more with many of the genome sequences representing that species. Use of the *Galleria mellonella* killing assay showed that C-214 was pathogenic in this model infection system. The strain C-214 had a colistin and polymyxin B MIC of 32 and 16 mg/L, respectively. Besides, it was resistant to cefotaxime, amikacin, and tetracycline and showed moderate biofilm-producing ability. Different genes associated with virulence or resistance to major classes of antibiotics were detected. We observed mutations in *lpxA*/*C*/*D* in C-214 and other *A. colistiniresistens* strains as probable causes of colistin resistance, but the biological effects of these mutations require further investigation. This study provides genomic insights into *A. colistiniresistens*, a potentially pathogenic bacterium isolated from a community member and notes the public health threat it may pose.

## Introduction

The development of antibiotic resistance in bacteria has increased greatly over time and poses a significant risk to public health. The abuse of antibiotics in human and veterinary medicine, agriculture, and poultry production contributes to the emergence of microorganisms resistant to antibiotics. In addition to being found frequently in healthcare facilities, multidrug-resistant bacteria are increasingly being identified in the community and surroundings environmental sources^1–3^.

Among antibiotic-resistant bacteria, *Acinetobacter* spp*.* have emerged as opportunistic pathogens often related to healthcare-associated infections^4,5^. However, diverse species of *Acinetobacter* have been isolated from different sources. Even though *A. baumannii* is unequivocally clinically and epidemiologically the most important *Acinetobacter* species, other *Acinetobacter* species have also been linked to human infections and found to be antibiotic-resistant and able to spread among hospitalized patients^6–8^. A study by Touchon et al.^9^ revealed that the *Acinetobacter* genus consists of isolates whose core DNA sequences are surprisingly variable and they identified a clade containing members with proteolytic or hemolytic activity. Seven of these members were named as species and six others as genomic species, including one named 13BJ/14TU^9^. Nemec et al.^10^ investigated the taxonomic status of 40 isolates of *Acinetobacter* and named an additional five species. In 2017, Nemec et al.^11^, investigated the genomic species 13BJ/14TU and found 24 strains with characteristic *rpoB/gyrB* sequences. These sequences had all been isolated from patients and had high levels of colistin (polymyxin E) resistance that is not observed in any other species within the hemolytic/proteolytic clade^6,12^. Due to the intrinsic resistance to colistin, Nemec et al.^11^ renamed the 13BJ/14TU genomic sequence isolate as *Acinetobacter colistiniresistens*. The genome assembly of the 13BJ/14TU isolate is available as GCF_003227755.1.

The genus *Acinetobacter* is a strictly aerobic, gram-negative coccobacilli with oxidase-negative and catalase-positive characteristics^11^. So far, the species *A. colistiniresistens* has been isolated only from clinical specimens, including sputum^5^, skin, blood^13^, vagina, eye, wound swab, catheter, conjunctiva and cerebrospinal fluid^11^. Its presence is typically linked to severe illnesses such as septicemia^14,15^. *A. colistiniresistens* type strain NIPH 2036^T^ (genome assembly GCF_000413935.1) was isolated prior to 1990 from a catheter in Belgium^13^. There is no evidence of characterization of this species from environmental niches, animals or healthy individuals.

The emergence and prevalence of multidrug-resistant (MDR) *Acinetobacter* spp has led to the reintroduction of the polymyxin antibiotic colistin as a first-line therapy for such infections^12,16^. Consequently, colistin resistance in *Acinetobacter,* and other bacteria, has emerged worldwide, including in Malaysia, reducing the options for treating MDR pathogens. Two mechanisms for colistin-resistance have been described in *A. baumannii*: (1) The addition of phosphoethanolamine to the lipid A part of lipopolysaccharide (LPS), which is caused by mutations in the genes encoding signalling proteins *PmrA* and *PmrB*, and (2) the loss of LPS production, which is caused by mutations in the *lpxA*, *lpxC* and *lpxD* genes^17^. However, there are no detailed studies on the colistin resistance in *A. colistiniresistens* strains. Additionally, no virulence-related study has been reported yet on *A. colistiniresistens.*

In light of the growing clinical significance of *A. colistiniresistens* and its heightened antibiotic resistance, understanding its potential reservoirs and routes of exposure has become a pressing concern. However, the transmission and genotypic characteristics of *A. colistiniresistens* within the community remain poorly understood. Here we report the characterization of an *A. colistiniresistens* isolated from the feces of a healthy individual. To the best of our knowledge, this is the first study of *A. colistiniresistens* isolated from the community. We phenotypically and genotypically characterized the isolated *A. colistiniresistens* strain and conducted a comparative whole genome sequence analysis with other *A. colistiniresistens* strains curated from NCBI. The larvae of the greater wax moth, *Galleria mellonella*, a relatively simple, non-mammalian model, was used to explore the pathogenicity of the *A. colistiniresistens* strain^18^. The data generated from this study provides insights into the genetic diversity within *A. colistiniresistens* strains and highlights its potential threat to the community.

## Methods

### Ethics approval

The study was approved by the ethics committee/IRB of Monash University Human Research Ethics Committee (MUHREC, project number: 1516), which is in accordance with the WMA Declaration of Helsinki (WMA and World Medical Association 2013). Written informed consent was obtained from each participant involved in the study. In addition, this study was performed in collaboration with the Southeast Asia Community Observatory (SEACO) based in the Segamat District of the southern Johor state in Peninsular Malaysia.

### Sample collection and Isolation of *Acinetobacter* spp

A single colistin-resistant *Acinetobacter* isolate was obtained after screening 233 fecal samples from Segamat for bacterial and fungal isolates in 2018. The isolate formed part of a larger cohort of *Acinetobacter* spp. isolates identified during a community research project that involved the isolation and investigation of ESKAPE pathogens from individuals living in the Segamat District^19^.

The sample collection and processing steps have been described earlier^20^. The samples were plated on Leeds Acinetobacter Agar (HiMedia, India) and MacConkey Agar (Oxoid, UK) and subsequently incubated at 37 °C for 24 h. Colony morphology and nature of the strains were observed and recorded. Three colonies with *Acinetobacter* morphology were selected from each sample and identified by standard biochemical methods (Gram stain, Catalase test and Oxidase reactions).

PCR amplification of a 16S rRNA gene fragment and subsequent sequencing was performed to confirm the *Acinetobacter* spp. The 16S rRNA gene was targeted using the universal primers described in previous studies^21^. Bacterial DNA extraction for PCR was carried out by the boiling extraction method described by Dashti et al.^22^.

### Colistin and other antimicrobial susceptibility testing

Antimicrobial susceptibility testing and interpretation were performed using the standard disk diffusion method for 12 different antibiotics on Mueller Hinton agar (Oxoid, UK) according to the Clinical Laboratory Standards Institute (CLSI) guidelines^23^. The antibiotic disks used in this study were piperacillin, piperacillin-tazobactam, ceftazidime, cefepime, aztreonam, imipenem, meropenem, gentamicin, amikacin, ciprofloxacin and tetracycline.

However, broth microdilution was used for colistin and polymyxin B, the only method CLSI recommends. The minimum inhibitory concentration (MIC) for colistin and polymyxin B was performed following the CLSI, 2015 guidelines and observed breakpoints (≤ 2 mg/L, susceptible; > 4 mg/L, resistant). *Acinetobacter baumannii* ATCC BAA 1605 and *E. coli* ATCC 2325 were used as controls with known antibiotic resistance patterns.

### Biofilm production and quantification assay

Biofilm production and quantification assays were performed according to Huet et al.^20^ with slight modifications. In brief, a total of 100 μl Tryptone Soya Broth (TSB) medium (Oxoid, UK) supplemented with 0.2% glucose was added to each well. Using an overnight bacterial culture, the cell suspension was adjusted to 0.5 McFarland standard in TSB supplemented with 0.2% glucose and 100 μl of each suspension was inoculated into each well. Two wells were left uninoculated and used as negative controls. The plates were incubated at 37 °C for 24 h for biofilm production. Following the biofilm production assay, biofilm quantification was carried out using Crystal violet (CV) and XTT assays.

### DNA extraction and whole genome sequencing

To achieve the complete genome sequence, hybrid short and long-read based whole genome sequencing (WGS) was performed. Total genomic DNA was extracted using the phenol–chloroform phase-separation method, according to Sambrook & Russell^24^. Extracted DNA quality and concentration was assessed using a Nanodrop bioanalyzer spectrophotometer (Thermo Scientific, Delaware, USA).

The short-read sequencing data was generated with a Nextera XT library preparation kit (Illumina, San Diego, CA, USA) and sequencing was performed using an Illumina MiSeq sequencer with the MiSeq Reagent Kit v3 as per the manufacturer's protocol (2 × 250 bp paired-end read setting). Additionally, for long-read sequencing, DNA libraries were prepared according to the Ligation Sequencing Kit protocol (SQK-LSK109). The long-read sequencing data was then generated using a MinION FLO-MIN106 flow cell and a MinION MK1B sequencing device (Oxford Nanopore Technology).

### De novo genome assembly and annotation

Quality trimming and filtering of the raw MiSeq short-reads were performed using Trimmomatic—0.39 version with the parameters PE, ILLUMINACLIP: adapters/NexteraPE.fa:2:30:10:8, LEADING:3, TRAILING:3, SLIDINGWINDOW:5:20, MINLEN:35 (Bolger et al.^25^). The short read draft genome was de novo assembled using SPAdes 3.13.0^26^. For the long read, base-calling was conducted using Guppy v3.2.10 through MinKnow v3.6.17, using the fast base calling configuration. The long-read genome assembly was performed using Flye v2.7^27^ and the sequence was later corrected and polished with the trimmed short-reads using Pilon v1.23^28^. The quality of the corrected assembly was evaluated using BUSCO v4.0.6^29^. Functional annotation was performed using Prokka 1.13^30^ and the genome map was plotted using BLAST Ring Image Generator (BRIG) v0.95^31^. Plasmids were detected using Plasmid Seeker^32^.

### Precise species identification

Species identification was carried out through the average nucleotide identity (ANI) based on BLAST and in silico DNA-DNA hybridization (isDDH) using the online server tool JSpeciesWS^33^ and genome-to-genome distance calculator^34^, respectively, with default parameters. An ANI value of more than 95% and isDDH values ≥ 70.0% were used as a cut-off to define bacterial species precisely. A phylogenomic analysis of closely related *Acinetobacter* spp. whole genome sequences was carried out using GToTree program v.1.7.05^35^. These sequences were retrieved from the National Center for Biotechnology Information (NCBI) based on the presence of single copy genes in each genome, including our isolate C-214.

### Genome analysis

A comparative genomic analysis was carried out between our strain C-214 and the genome sequences of 20 other *A. colistiniresistens* strains obtained from NCBI (GCF_000248195.1, GCF_000369645.1, GCF_000369765.1, GCF_000413935.1, GCF_000876115.1, GCF_003227755.1, GCF_003569565.2, GCF_007713425.1, GCF_008982465.1, GCF_008984005.1, GCF_008987005.1, GCF_008988385.1, GCF_008990765.1, GCF_008992365.1, GCF_008993755.1, GCF_009013055.1, GCF_009013115.1, GCF_009013295.1 and GCF_900406805.1). Two *A. baumannii* strains (H-10112 and C-98) collected from the same location during the study were used to compare the sequence variation with *A. colistiniresistens* strains. The strain H-10112 was an MDR hospital strain, and C-98 was a non-MDR community strain^36^.

The comprehensive antibiotic resistance database (CARD)^37^ was used to identify acquired antibiotic-resistance genes using Abricate version 1.0.1(https://github.com/tseemann/abricate). Virulence-associated genes were identified using the virulence factor database (VFDB4)^38^. Mobile genetic elements were detected using ISFinder^39^. Gene content matrices were obtained using anvi’o^40^.

An *ampC* gene was detected in the genome of the C214 isolate. It resembles the UniRef90_N9PW73 cluster (UniRef50_A0A0N1I997 cluster at 50% cutoff), whose protein sequences belonged exclusively to *A. colistiniresistens*. The AmpC protein sequence from C214 was compiled together with protein sequences of the UniRef50_A0A0N1I997 cluster and Ambler class C beta-lactamases from the BLDB database^41^ to build a phylogenetic tree using FastTree^42^. The *ampC* gene tree was visualized using iTol v6^43^.

### In vivo *Galleria mellonella* killing assay

To determine the virulent nature of *A. colistiniresistens*, an in vivo killing assay was performed on the greater wax moth, *Galleria mellonella*. The *G. mellonella* larvae were purchased from Carolina Biological, US. Larvae showing symptoms of melanization or deformation were omitted from the assay to eliminate the potential for bias. Each larva was weighed and those meeting the criteria of 250 ± 50 mg were used in the study. Killing assay experiments were performed by injecting 10 µl of two different bacterial solutions with 10^7^ and 10^6^ colony-forming units per larva (CFU/larva), respectively, into the last left proleg using a Hamilton syringe. To check for death caused by physical damage, one group of larvae was injected with 10 µl of PBS as a negative control. Another control group did not receive an injection. The larvae were incubated for seven days at 37 °C and checked for symptoms of death every 24 h. Larva that did not respond to tactile stimulation or had a blackish discolouration were reported dead. *A. baumannii* C-98 and *E. coli* OP50 were selected as high and low-pathogenicity reference strains, respectively. The experiments were repeated three times, with the average reading taken into account.

### Statistical data analysis

All analyses were performed using three separate experiments using GraphPad Prism software 6.01. The significance of differences was determined at *p* ≤ 0.05. The killing of *G. mellonella* by *A. colistiniresistens* was analyzed using the Kaplan–Meier method. Log-rank test was performed.

## Results

### Characteristics of the isolate

A study of *A. baumannii* from fecal samples from the community in Segamat district, Johor, Malaysia led to the isolation of a single colistin-resistant *Acinetobacter* spp*.* designated as C-214, on selective agar plates. The carrier was a 34-year-old female housewife from the indigenous Orang Asli Jakun community.

For preliminary species identification, PCR was done with the universal primers 27F and 1492R followed by Sanger sequencing to get the nearly complete 16S rRNA gene sequence of the strain^36^. The use of BLAST for the 16S rRNA sequence against the NCBI database revealed that the isolate belonged to the genus *Acinetobacter* and is a member of the species *colistiniresistens*.

FE-SEM imaging was carried out, and the colony morphology was compared to explore any differences in bacterial cell morphology between *A. baumannii* and *A. colistiniresistens*. No significant difference was observed in their cell membrane and colony formation. Both were found to have coccobacillus phenotypes. On selective Leeds *Acinetobacter* agar media, they produced identical colonies and colours (data not shown).

### Antibiotic resistance profile and biofilm-forming ability

The isolate, C-214, had a colistin and polymyxin B MIC of 32 and 8 µg/ml, respectively (Table 1). In addition to colistin resistance, this isolate was phenotypically resistant to cefotaxime, amikacin and tetracycline but susceptible to cefepime, ceftazidime, ciprofloxacin, gentamicin, piperacillin/tazobactam and carbapenems based on the disk diffusion antibiotic susceptibility testing (Table 1).

**Table 1:** Antibiotic resistance profile and biofilm formation of strain C-214.

Strain: C-214	Antibiotics	Interpretive categories, zone diameter, breakpoints			Results zone diameter (mm), breakpoints	
		S	I	R		
AST	PRL 100	≥ 21	18–20	≤ 17	21	S
	TZP 110	≥ 21	18–20	≤ 17	26	S
	SAM 20	≥ 15	12–14	≤ 11	18	S
	CAZ 30	≥ 18	15–17	≤ 14	18	S
	CTX 30	≥ 23	15–22	≤ 14	13	R
	FEP 30	≥ 18	15–17	≤ 14	23	S
	IPM 10	≥ 22	19–21	≤ 18	30	S
	MEM 10	≥ 18	15–17	≤ 14	27	S
	CN 10	≥ 15	13–14	≤ 12	20	S
	AK 30	≥ 17	15–16	≤ 14	12	R
	CIP 5	≥ 21	16–20	≤ 15	28	S
	TE 30	≥ 15	12–14	≤ 11	10	R
MIC	Colistin (µg/ml)	≥ 2	–	≤ 4	32	R
	Polymixin B (µg/ml)	≥ 2	–	≤ 4	16	R
Biofilm	XTT	Moderate				
	CV	Moderate				

*C* community isolates; *R* resistant; *S* susceptible; *I* intermediate; *PRL100* piperacillin 100 µg; *TZP110* piperacillin-tazobactam 110 µg; *SAM20* ampicillin/sulbactam 20 µg; *CAZ30* ceftazidime 30 µg; *CTX30* Cefotaxime 30 µg; *FEP30* cefepime 30 µg; *IPM10* imipenem 10 µg; *MEM10* Meropenem 10 µg; *CN10* gentamicin 10 µg; *AK30* Amikacin 30 µg; *CIP5* ciprofloxacin 5 µg; *TE30* tetracycline 30 µg.

The biofilm-forming ability of C-214 was also assessed. Based on XTT and CV assays, isolate C-214 showed moderate biofilm-forming capability (Table 1).

### Genomic features of the strain C-214

**Figure 1:**
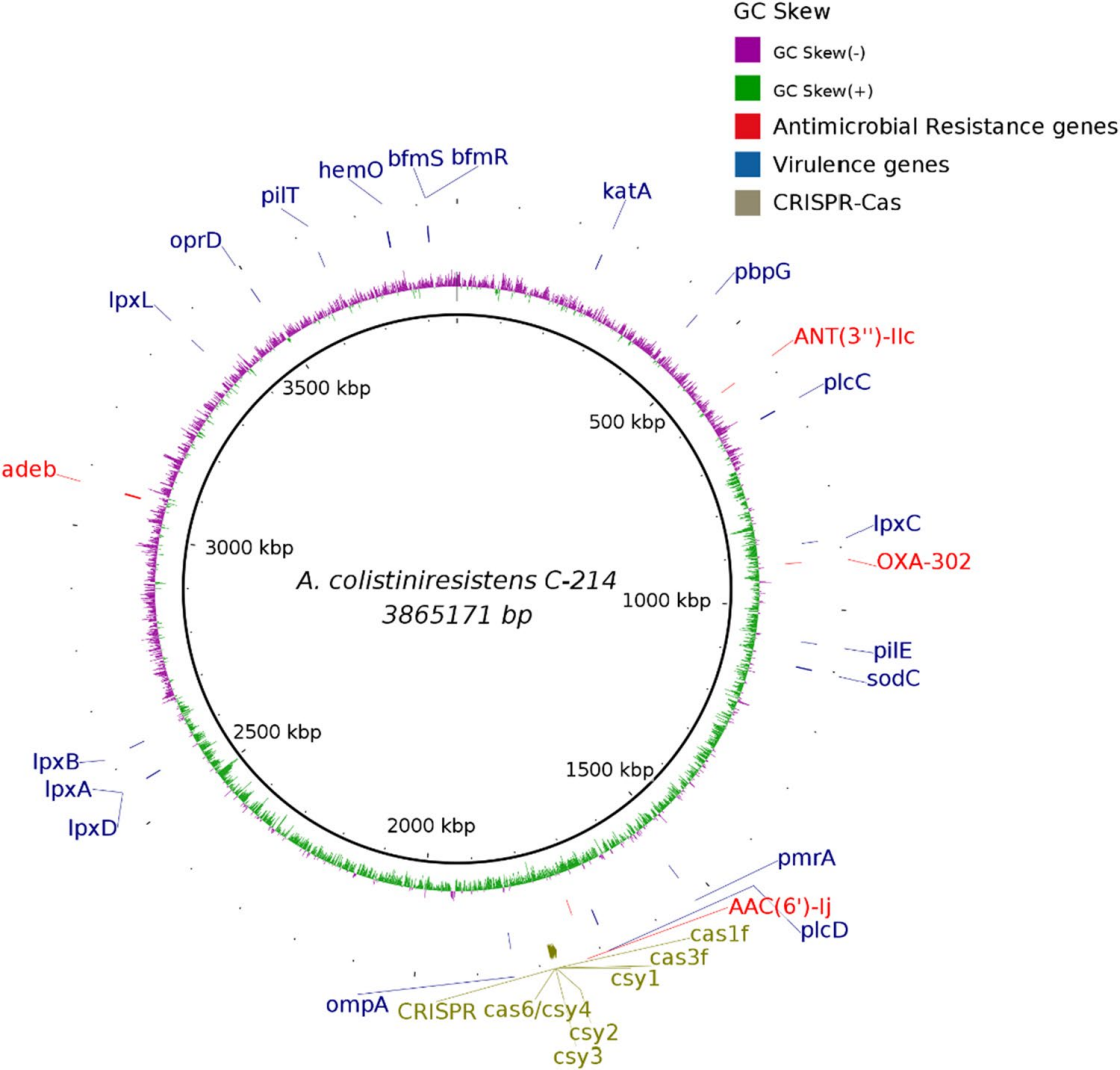
Genome map of *Acinetobacter colistiniresistens* strain C-214 chromosome (CP102099) plotted using BLAST ring image generator (BRIG). The outer coloured circle denotes the GC skew of genomic sequences (purple: negative; green: positive), followed by distributions of antimicrobial resistance genes (red), virulence genes (blue) and CRISPR-Cas system loci (grey).

C-214 was sequenced using both short-reads (Illumina MiSeq) and long-read (Oxford Nanopore) sequencing technologies. Hybrid genome assembly revealed that the *Acinetobacter* strain C-214 contained one circular chromosome of 3,865,171 bp (GC content 41.33%) (Fig. 1). The GC content is almost identical to that reported for the *A. colistiniresistens* sequence represented by GCF_003227755.1 and the genome size is typical for this strain. Besides, three circular plasmids were also detected with sizes of 10,411 bp (p214-1), 5509 bp (p214-2), 8305 bp (p214-3) and GC content of 35.4, 30.79 and 33.65%, respectively (Supplementary B). The average genome size is similar to *A. baumannii*, whose genomes range between 3.6 and 4 Mbp in size with a GC content of around 39%^44,45^.

The genome annotation analysis using Prokka detected 3844 genes with 3,705 coding sequences, 75 tRNA sequences, 18 rRNA sequences, 1 tmRNA sequence and 45 misc RNA sequences.

### Precise species detection

**Figure 2:**
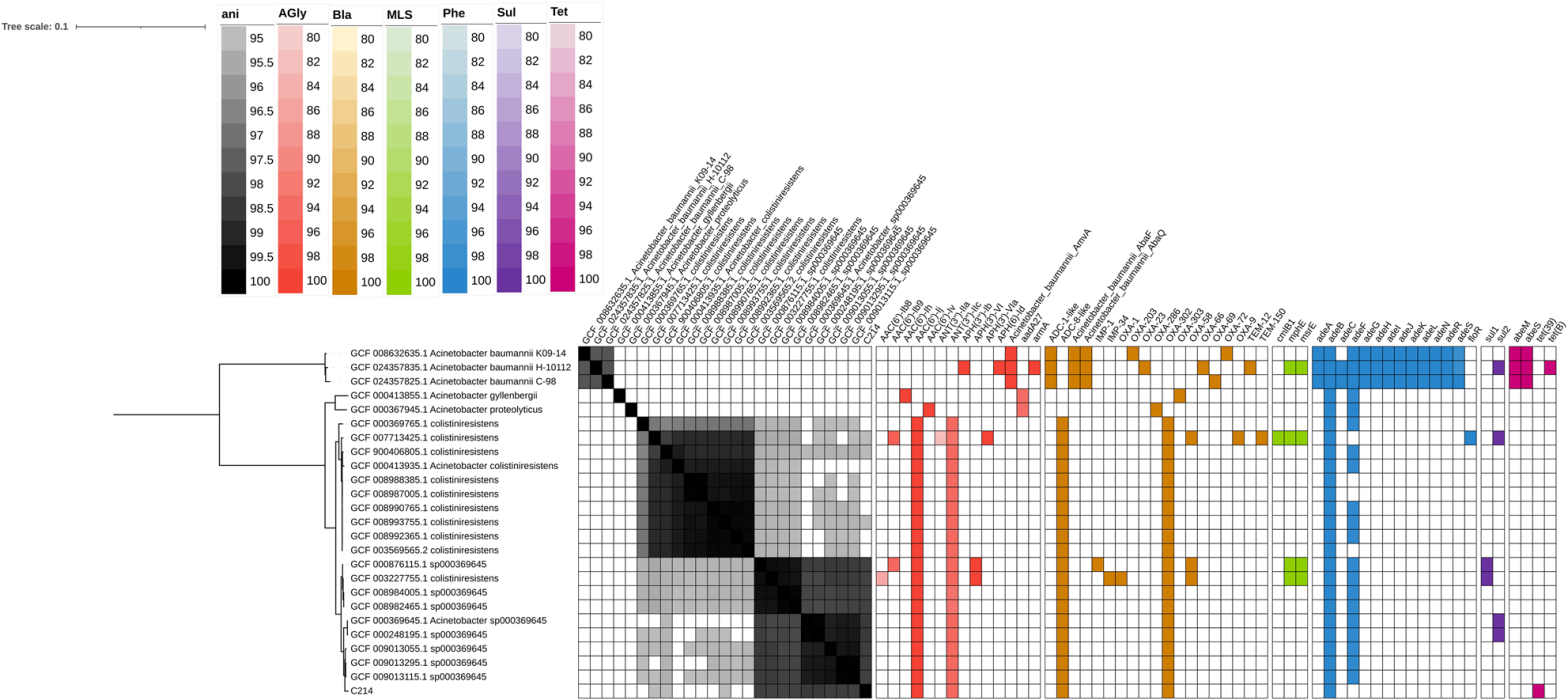
The phylogenomic tree of *Acinetobacter colistiniresistens*, annotated with ANI values and percentage identity matrix against antimicrobial resistance genes from CARD. Note that the type strain NIPH2036 is represented by the genome assembly GCF_000413935.1, the 13BJ/14TU isolate by GCF_003227755.1 and three TUM isolates by GCF_009013115.1, GCF_9013295.1 and GCF_9013055.1.

ANI and in silico DNA-DNA hybridization analyses of strain C-214 were conducted against 79 different *Acinetobacter* spp along with 19 *A. colistiniresistens* strains (Supplementary B). The highest ANI (98.08%) and DDH (71.04) values were found against *A. colistiniresistens* strain NR1165 (Supplementary B). A phylogenetic tree was constructed on the basis of 20 *A. colistiniresistens,* three *A. baumannii,* one *A. gyllenbergii* and one *A. proteolyticus* genomes, and it is clear that C-214 is a genomovar within the *A. colistiniresistens* group which is itself composed of two sub-groups (Fig. 2).

### Presence of antibiotic resistance genes in C-214 compared with other *A. colistiniresistens* strains

The distinct patterns of antibiotic resistance phenotypes observed in strain C-214 prompted us to investigate the known genes linked to resistance in the sequenced genome and compare them with other available *A. colistiniresistens* genomes. In addition, we also compared the presence of AMR genes in three *A. baumannii,* one *A. gyllenbergii* and one *A. proteolyticus* genomes. Detailed results are summarized in Fig. 2. The AMR gene analysis using the CARD database detected five antibiotic resistance genes in the genome of strain C-214, where one tetracycline resistance gene (*tet*39) was found within a plasmid (p214-1). Besides, one beta-lactam resistance gene *bla*_OXA302_, two aminoglycoside resistance genes (*ANT(3’’)-IIc*, *AAC(6’)-Ij*) and multidrug efflux pump gene *adeB* were also present in the isolate. Detection of these genes also supported our AST phenotypic data (Table 1), where the strain C-214 showed resistance against beta-lactams (CTX-30), tetracycline (TE30) and aminoglycoside (AK30). However, even though the isolate is highly resistant against colistin and polymyxin B, the gene responsible for resistance could not be identified. Studies have found that the plasmid-mediated gene *mcr* is responsible for colistin resistance in *Acinetobacter baumannii*^46^. We could not find any *mcr* gene in any *A. colistiniresistens* isolates.

**Figure 3:**
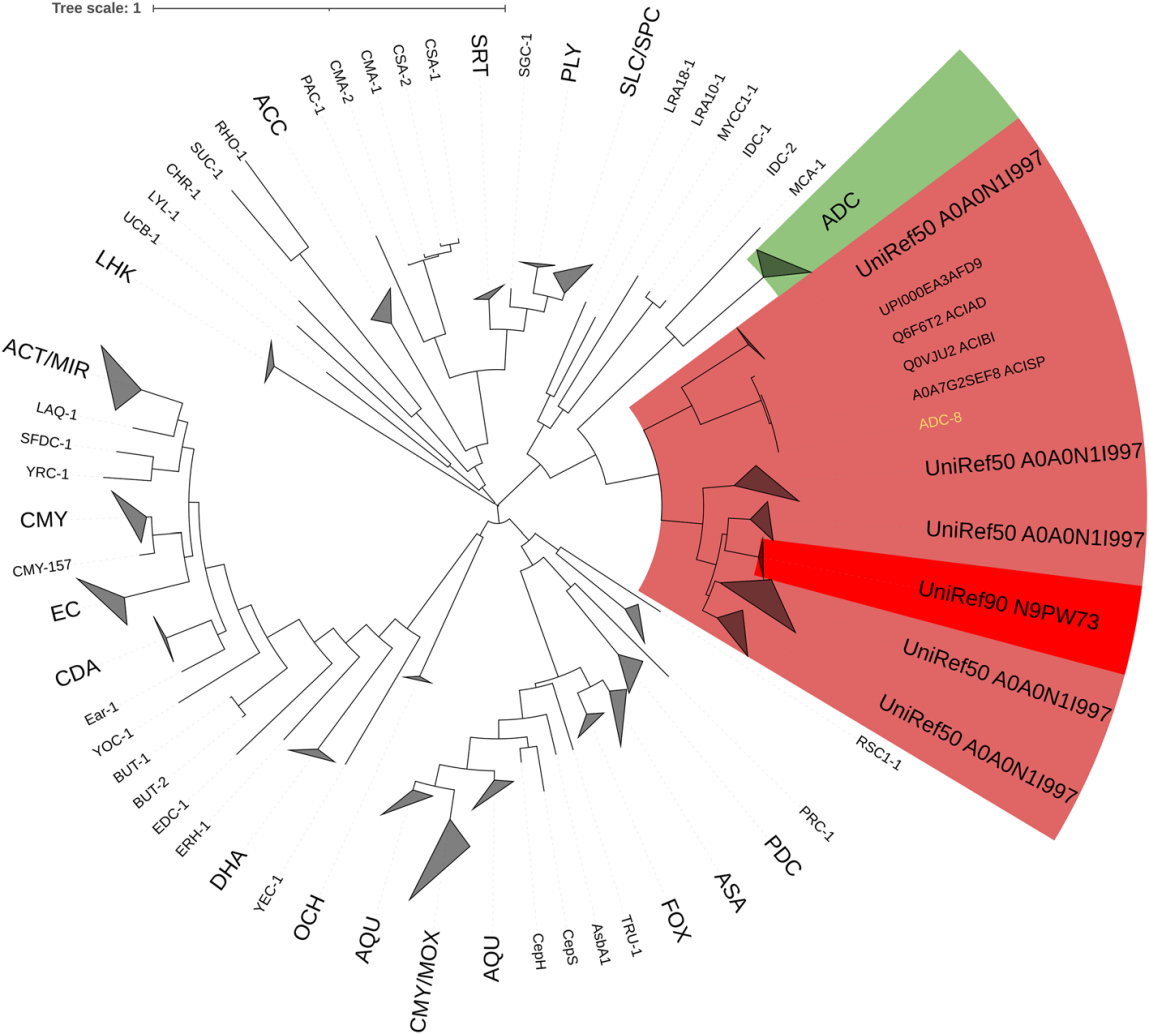
Phylogenetic tree based on curated Ambler class C beta lactamases from BLDB and UniRef90_N9PW73 and UniRef50_A0A0M1I997 sequences. AmpC sequence from C-214 clusters together with UniRef90_N9PW73 sequences. In green are the *Acinetobacter* derived cephalosporinases (ADCs) except ADC-8. The brown group is a clade consisting of UniRef50_A0A0M1I997 sequences. UniRef50 sequences are sequences that form a 50% similarity cluster (50% is supposedly a lenient cutoff here). We can also see that the UniRef50_A0A0M1I997 cluster form two subclades. The C-214 *ampC* gene (UniRef90_N9PW73 "sub-"subclade, red) does not fall under the ADC-8 subclade. Here, the three-letter names refer to different beta-lactamase’s which are all class C.

After comparison of the putative resistance genes in C-214 with 19 other *A. colistiniresistens* strains, it was discovered that most of the isolates carried a similar collection of resistance genes (n ≤ 5). However, the tetracycline resistance A gene (*tet39*) was only detected in the strain, C-214, described in this paper. An insertion sequence *ISaba26* was detected in both the chromosome and plasmid (p214-1). In addition, when *Acinetobacter* derived cephalosporinases (ADCs) were compared between *A. colistiniresistens* isolates and *A. baumannii*, considerable differences were observed. Both *A. baumannii* and the *A. colistiniresistens* isolates carried the class C intrinsic beta-lactamase gene. However, whereas the *A. baumannii* isolates analyzed in this study carried an ADC-1 type of gene, the *A. colistiniresistens* carried an ADC-8 type gene (Fig. 3). The amino acid sequence similarity of these two types is about 50%. Efflux pump related genes were commonly seen in both *A. baumannii* strains, but only two genes were found in the *A. colistiniresistens* isolates.

### Virulence factor related genes observed in *A. colistiniresistens*

**Figure 4:**
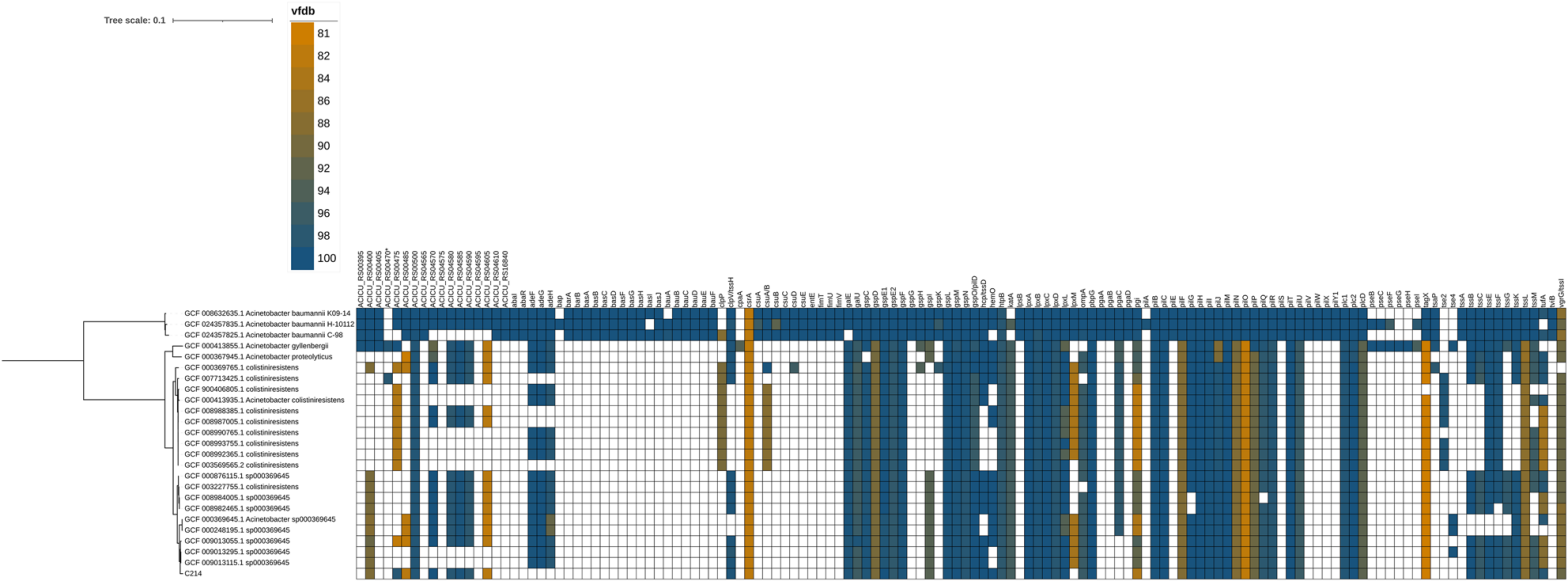
Presence of genes involved in virulence in the strain C-214 and 19 other *A. colistiniresistens*, three *A. baumannii,* one *A. gyllenbergii* and one *A. proteolyticus* genomes. The presence of genes in an isolate is specified by a colored rectangle, colored according to sequence similarity to curated VFDB sequences. The absence of genes is shown as blank spaces with no color.

Different virulence factor related genes were analyzed in the C-214 strain, along with 19 other *A. colistiniresistens,* three *A. baumannii,* one *A. gyllenbergii* and one *A. proteolyticus* genomes derived from NCBI, where two *A. baumannii* genomes from the same project. The results are summarized in Fig. 4. The outer membrane protein gene *ompA,* which promotes bacterial biofilm formation, eukaryotic cell infection, antibiotic resistance, and immunomodulation, was found in all *A. colistiniresistens* isolates, including C-214^47^. Genes related to lipopolysaccharide (LPS) production, such as *lpxA*, *B*, *C*, *D* and *lpxL,* were present in all *A. colistiniresistens* genomes. The *lpxA*, *lpxC*, and *lpxD* genes are primarily involved in the initial stages of lipid A production and the hydrophobic anchor of LPS^48^. It has been found that mutations in *lpxA*, *lpxC* and *lpxD* may play a role in the development of colistin resistance^48^. We compared these *lpx* genes acquired from WGS of all *A. colistiniresistens* and three *A. baumannii* colistin-sensitive strains (*A. baumannii* ATCC19606 type strain, *A. baumannii* H-10112, *A. baumannii* C-98) (Supplementary A, Fig. S1). We found similar polymorphisms in *lpxA*/*C*/*D* and *lpxL* genes in all *A. colistiniresistens* strains suggesting that alterations in LPS metabolism could be the reason for colistin resistance observed in these strains. We also found other virulence factor related genes including; type VI secretion system, stress adaptation, antiphagocytosis, two-component regulatory system (*bfmR, bfmS*), serum resistance, iron uptake and adherence genes. While most isolates shared similar virulence-related genes (Fig. 4), certain differences were noted between *A. colistiniresistens* and *A. baumannii* isolates. Although both hospital and community *A. baumannii* isolates possessed a complete set of type VI secretion system genes, only one to three genes encoding this system were found in six *A. colistiniresistens* isolates, including C-214. Type VI secretion system (T6SS) genes are well recognized as a crucial virulence factor in *A. baumannii* and toxins produced by T6SS genes could kill other bacteria as well as eukaryotic cells^49^.

### Pangenome analysis

Extensive pan-genome analysis investigations can aid in understanding a bacterial species' functional adaptability^50^. To get insight into the pan-genome information of *A. colistiniresistens,* we created different plots to visualize the number of total genes, core accessory genes, and unique genes as a function of the sequenced genomes.

**Figure 5:**
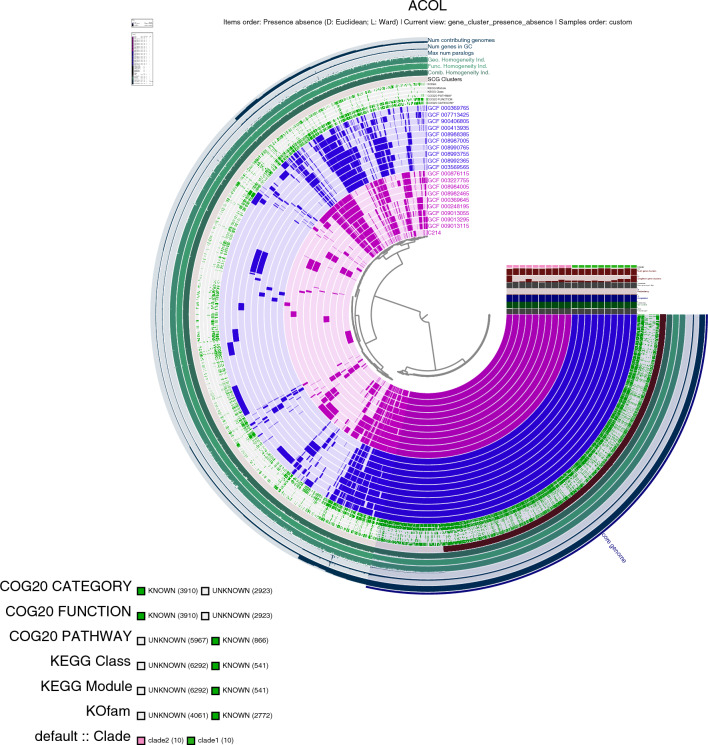
Anvi’o pangenome display of 20 *A. colistiniresistens* genomes. Layers were coloured according to the two proposed *A. colistiniresistens* genomovars. Genomes were sorted based on the phylogenomic tree from Fig. 2. Gene clusters were sorted based on the presence and absence of genes. Gene clusters that fall under the single copy genes and the core genome were indicated. Barplots representing singleton gene clusters per each genome were also shown.

*Acinetobacter colistiniresistens* is classified into two genomovars. To explore the genomic diversity, we performed a pan-genome analysis using the Anvi’o pan-genome workflow^40^ (Fig. 5). The pan-genome consisted of a total of 6825 genes, with 2601 core genes shared among all strains and 2179 core genes occurring only once. Furthermore, we investigated the enrichment between the two genomovars^51^. The enrichment was defined as an enrichment score greater than 15 and an adjusted q-value below 0.01. Functional enrichment analysis was conducted based on COG20 annotation (Supplementary B). Our strain, C-214, was found in clade 2, which exhibited enrichment in 17 genes, while clade 1 showed enrichment in eight genes. The genomes were arranged according to the phylogenomic tree (Fig. 5).

### Isolate pathogenicity

**Figure 6:**
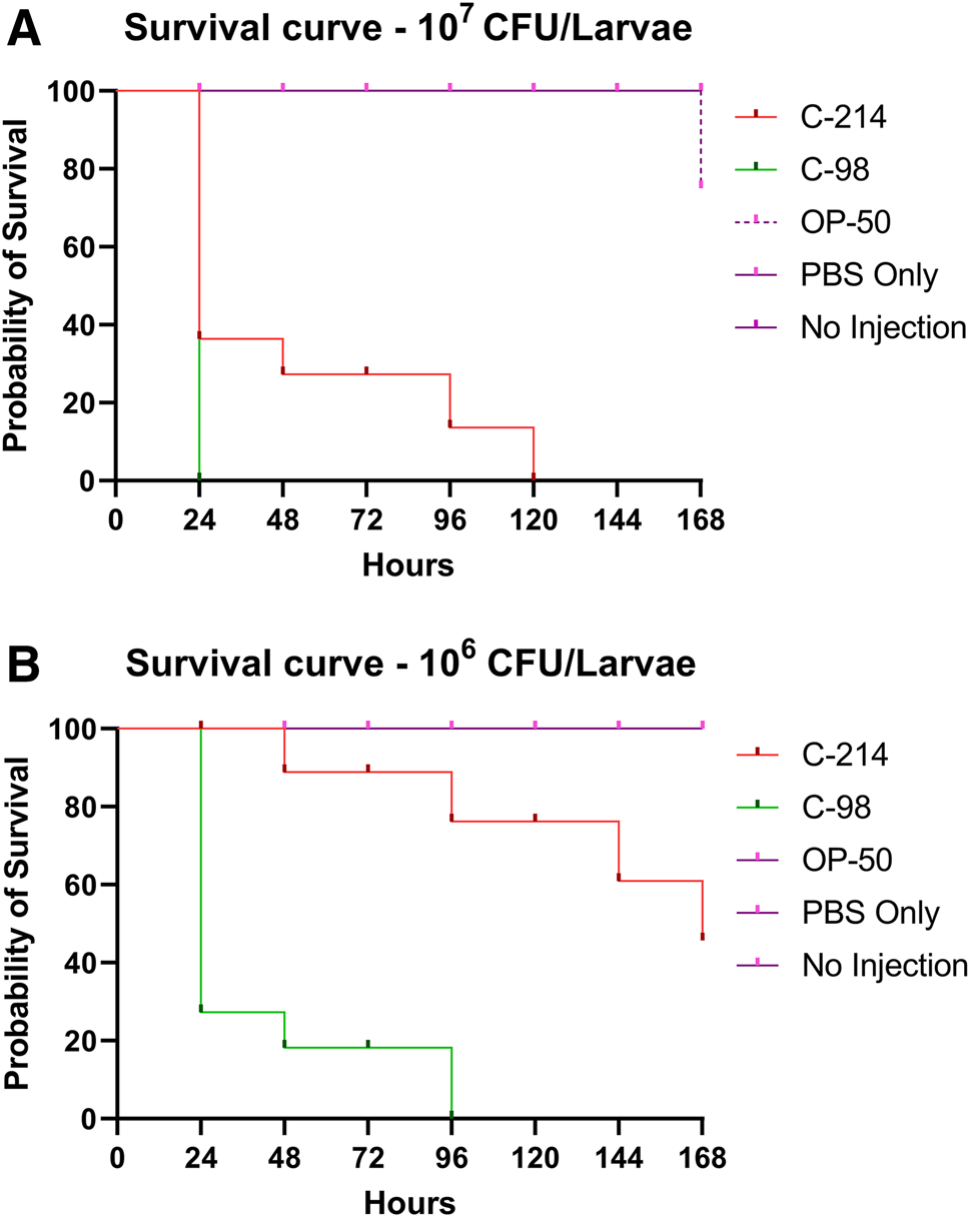
Kaplan–Meier survival distributions for dose-dependent challenges of *A. colistiniresistens* C-214. *A. baumannii* C-98 and *E. coli* OP50 were used as highly virulent and non-virulent strain controls, respectively, at all dosage levels. Three biological repeats of each experiment were pooled and results are shown as a percentage probability of survival. Infection results for all three isolates tested were significantly different (*p* = 0.001; Mantel–Cox log-rank test, demonstrating that larval survival is dependent on the quantity of bacteria injected.

The pathogenicity of *A. colistiniresistens* C-214 was tested in the *G. mellonella* model and compared using a virulent *A. baumannii* strain C-98 (unpublished) and a non-virulent *E. coli* OP-50. Figure 6 highlights the variability of pathogenicity in two different bacterial inoculums.

We administered parenteral injections of two different concentrations (10^7^ and 10^6^ CFU) of *A. colistiniresistens* strain C-214 to examine the impact on larval pathogenicity. The infected larvae exhibited distinct symptoms, including nodulation, blackening of the cuticle, and eventual mortality. Notably, the degree of melanization increased significantly with higher inoculum doses, indicating that the initial infectious inoculum size plays a crucial role in the progression of the infection. To analyze the survival outcomes, we employed Kaplan–Meier survival distributions for each bacterial inoculum and conducted a log-rank (Mantel–Cox) test, which revealed significant differences (*p* < 0.001). The survival probability of the larvae depended on the number of CFU injected. For larvae injected with an inoculum size of 10^7^ CFU/larvae, the survival rate after 24 h was 40% for *A. colistiniresistens* C-214, 0% for *A. baumannii* C-98, and 100% for the non-virulent *E. coli* strain OP50. However, the survival rate for C-214-treated larvae decreased to 0% after 120 h. In comparison, larvae injected with 10^6^ CFU/larva exhibited a 90% survival rate for C-214 after 24 h, 20% for C-98, and 100% for OP-50. Within the observation period of 168 h post-inoculation, 60% of the C-214-treated larvae survived. Further dilution (10^5^ CFU/larva) resulted in 100% survival but showed melanization in 30% of the population after 168 h of observation.

## Discussion

MDR *Acinetobacter baumannii* is a significant nosocomial pathogen that has been the primary focus of most research on *Acinetobacter* spp. Aside from *A. baumannii*, little is known about other *Acinetobacter* species. However, non-*baumannii Acinetobacter* species are increasingly being identified as causative agents of nosocomial infections. One such organism, *A. colistiniresistens* has been isolated from different sources, including sputum, blood, wound swab, catheter and conjunctiva in hospitalized patients^5,6,52^ (Supplementary B). However, the genomic features of this organism have been rarely discussed^11,52^. In this study, we focused on characterizing the *A. colistiniresistens* strain isolated from a healthy human fecal sample. This report represents a significant finding as it is the first known instance of this species being isolated from a healthy individual. The study offers new information on the genomic and virulence traits of *A. colistiniresistens*, which could be useful in treating this particular pathogen.

Treating infections caused by *Acinetobacter* spp. has become increasingly challenging due to their multidrug-resistance (MDR) and pan-drug-resistance (PDR) patterns. Colistin is commonly regarded as a last-resort antibiotic against MDR *Acinetobacter* infections. Hence, the presence of *A. colistiniresistens* within a healthy community, naturally resistant to colistin^11,12^, raises significant public health concerns. The C-214 isolate displayed high resistance to both colistin and polymyxin B, with minimum inhibitory concentrations (MIC) of 32 and 16, respectively. This resistance profile is comparable to that of the NR1165 isolate reported in a study conducted in Japan^5^, further exacerbating concerns regarding the spread of such resistant strains.

Baraka et al.^53^, identified resistance genes against sulfonamides, macrolides, ABC-F, and beta-lactamases antibiotics in previously isolated *A. colistiniresistens.* Similarly, WGS of *A. colistiniresistens* strain C-214 revealed several resistance genes, including beta-lactam resistance gene *bla*_OXA302_, tetracycline resistance *tet*39, aminoglycoside resistance *ANT(3'')-IIc*, *AAC(6')-Ij* which also supported our AST data. The presence of plasmids carrying genes such as *tet*39 makes this strain more threatening to the community as this might enable the strain to confer resistance genes to other species through horizontal gene transfer^54^.

A comparative WGS study with nineteen other *A. colistiniresistens* retrieved from NCBI revealed that most of the *A. colistiniresistens* isolates harboured similar types of AMR genes except two strains (NR1165, DL) carrying more AMR genes, including carbapenemase genes coding for OXA-58, IMP-34 and ESBL gene coding for TEM-181.

Though the *A. colistiniresistens* isolates shared some virulence properties of *A. baumannii*, there were some noticeable differences. For instance, the type VI secretion system gene number was lower in the *A. colistiniresistens* isolates. Though *A. baumannii* and *A. colistiniresistens* carry different subclasses of type VI secretion related genes, the number of genes lost in *A. colistiniresistens* could be of vital importance. Additionally, it has recently been shown that silencing the chromosomally encoded type VI secretion system is crucial for horizontal gene transfer by conjugation, which is essential for disseminating antibiotic resistance^55^. As such, the type VI secretion system in *A. colistiniresistens* warrants further investigation for its virulence and resistance properties. It is to be noted that although the individual who carried the *A. colistiniresistens* isolated in this study did not carry *A. baumannii,* such an occurrence is a possibility in the future. In such a situation, the transfer of additional resistances into *A. baumannii* or vice versa could lead to the organism being resistant to all currently used drugs. Consequently, infections by organisms like this would become difficult to treat.

Recently, non-animal in vivo models like *G. mellonella* have been used to determine the virulence of pathogens such *A. baumannii*, *P. aeruginosa*, *Burkholderia cepacia*, *Bacillus cereus*, and disease-causing fungi^18,56^. *G. mellonella* can tolerate incubation temperatures up to 37 °C, making it preferable for researching human illnesses^56^. It also breeds quickly and does not require animal ethics clearance. In our study, *G. mellonella* exhibited dose-dependently sensitivity to *A. colistiniresistens* (C-214) infection and might be utilized to research its pathogenicity.

In conclusion this study describes the first complete genome sequence of *A. colistiniresistens* strain isolated from the fecal sample of a healthy adult female individual from Malaysia. Salient genomic features of this strain included the presence of genes relevant to AMR and virulence. MDR *A. colistiniresistens* is an opportunistic pathogen and is naturally resistant to colistin, which is of great concern as it is an antibiotic of last resort. Further, In vivo *G. mellonella* killing assay indicated the pathogenic potential of the strain C-214. Carriage of *A. colistiniresistens* in the asymptomatic community poses a risk to public health, and more attention should be paid to epidemiological surveillance and transmission of this bacteria.

### Supplementary Information

Supplementary Figure S1.

Supplementary Information.

## Data Availability

The assembled genome sequence has been deposited in GenBank and NCBI database under Project number PRJNA863728. (The GenBank accession numbers for other *A. colistiniresistens* strains used for comparison are listed in Supplement B).
