# Supplementary material for: Genomic and phenotypic characterization of Acinetobacter colistiniresistens isolated from the feces of a healthy member of the community
Source: Sci Rep. 2023 Aug 3;13:12596. doi: 10.1038/s41598-023-39642-0 (PMC10400646; doi:10.1038/s41598-023-39642-0)
Supplement: Supplementary file 1 — Supplementary Figure S1. [file 41598_2023_39642_MOESM1_ESM.docx]

# **Supplementary Information:**

**Title: Emergence of Multi-Drug Resistant *Acinetobacter colistiniresistens* in a Healthy Human Gut: First Reported Case in Malaysia**

Nazmul Hasan Muzahid^1^*, Muhammad Zarul Hanifah Md Zoqratt^1^, Kah Ern Ten^1^, Md Hamed Hussain^1^, Tin Tin Su^2^, Qasim Ayub^1,3^, Hock Siew Tan^1,4^ and Sadequr Rahman^1,3,4^*

*^1^School of Science, Monash University Malaysia,* *Bandar Sunway, 47500, Selangor Darul Ehsan, Malaysia*

*^2^South East Asia Community Observatory (SEACO), & Global Public Health, Jeffrey Cheah School of Medicine & Health Sciences, Monash University Malaysia, Bandar Sunway, 47500, Subang Jaya, Selangor, Malaysia*

*^3^Monash University Malaysia Genomics Facility, Bandar Sunway, 47500, Selangor Darul Ehsan, Malaysia*

*^4^Tropical Medicine & Biology Multidisciplinary Platform, Monash University Malaysia, Bandar Sunway, 47500, Subang Jaya, Selangor, Malaysia*

## Corresponding author and email address:

Nazmul Hasan Muzahid: [nazmul.muzahid@monash.edu](mailto:nazmul.muzahid@monash.edu)

Sadequr Rahman: [sadequr.rahman@monash.edu](mailto:sadequr.rahman@monash.edu)

School of Science, Monash University Malaysia, Jalan Lagoon Selatan, 47500 Bandar Sunway, Selangor Darul Ehsan, Malaysia


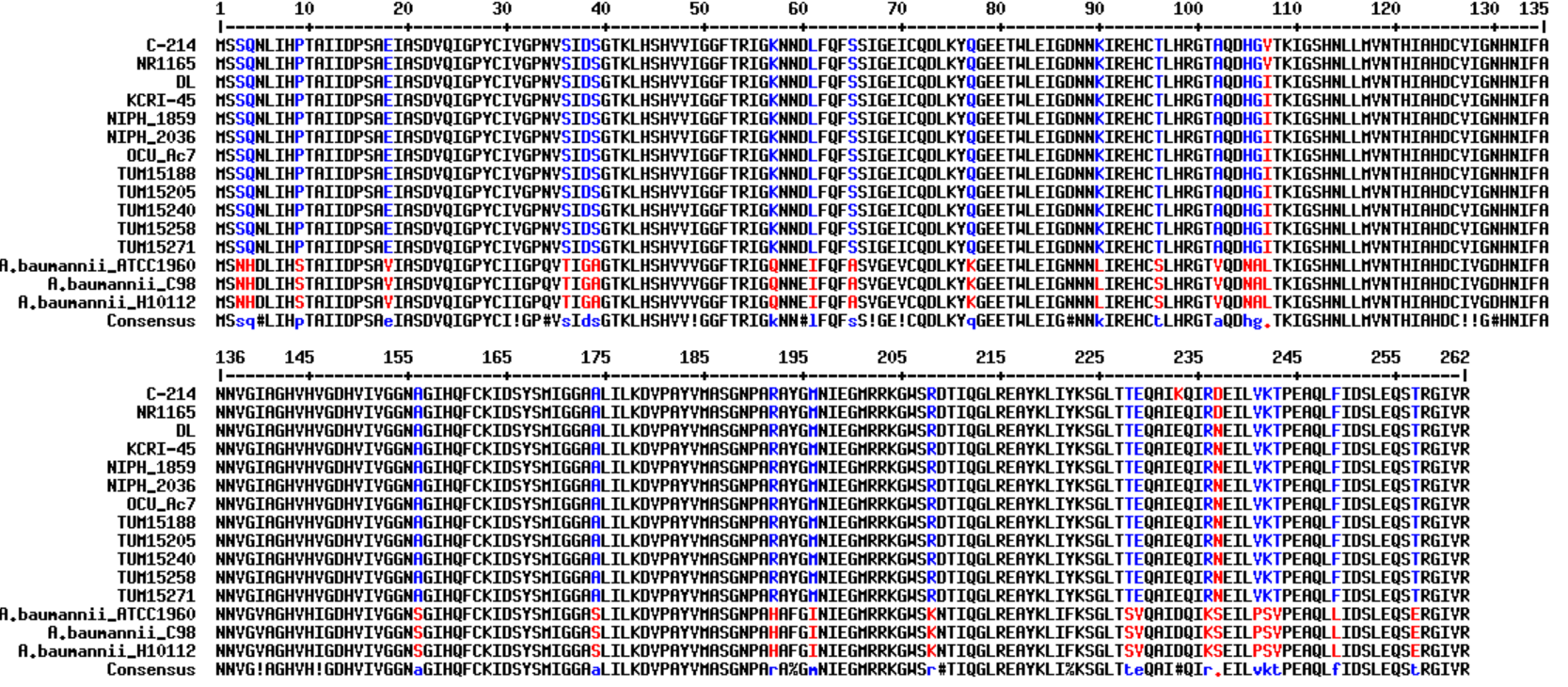
*lpxA*


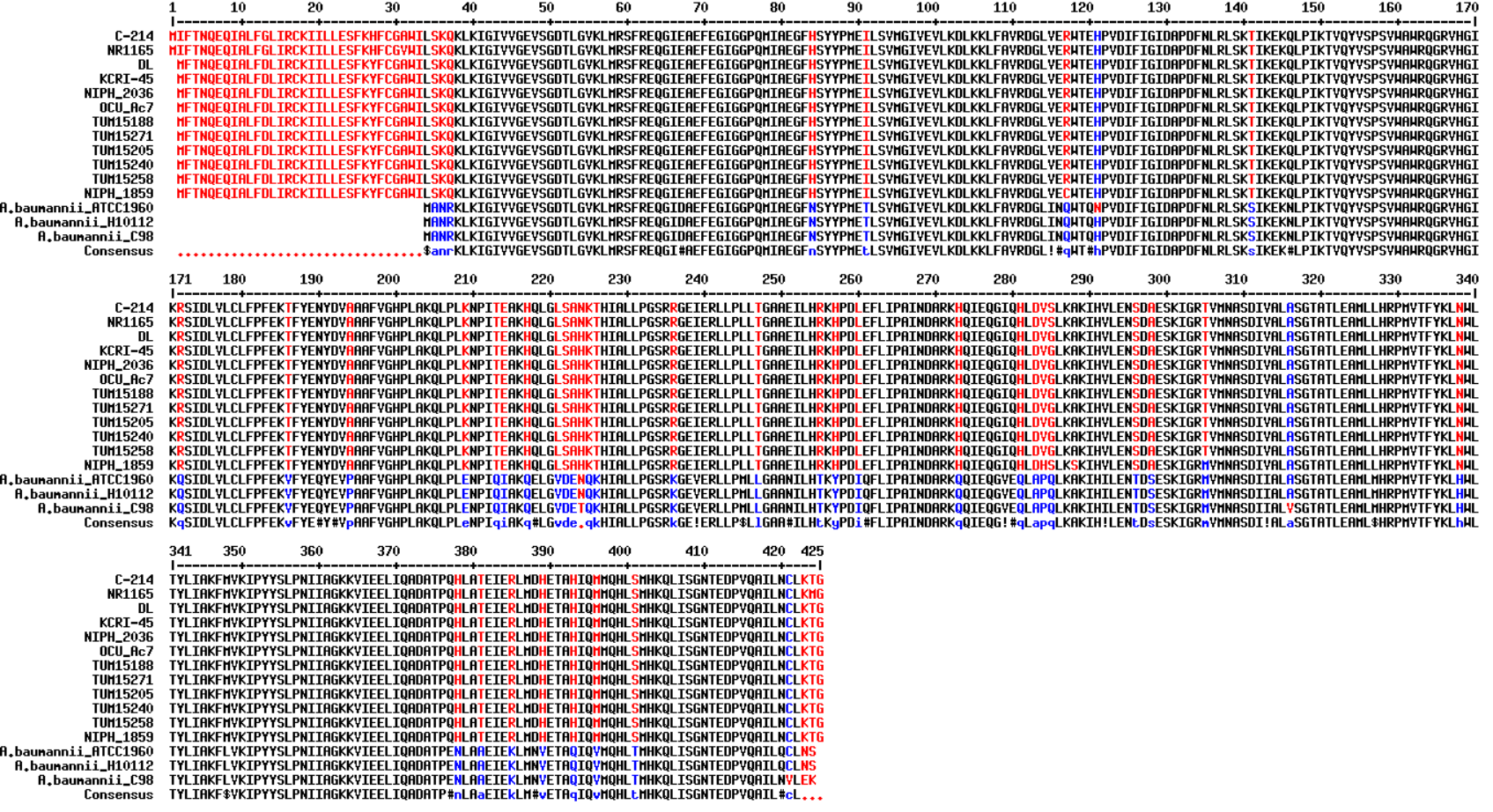
*lpxB*


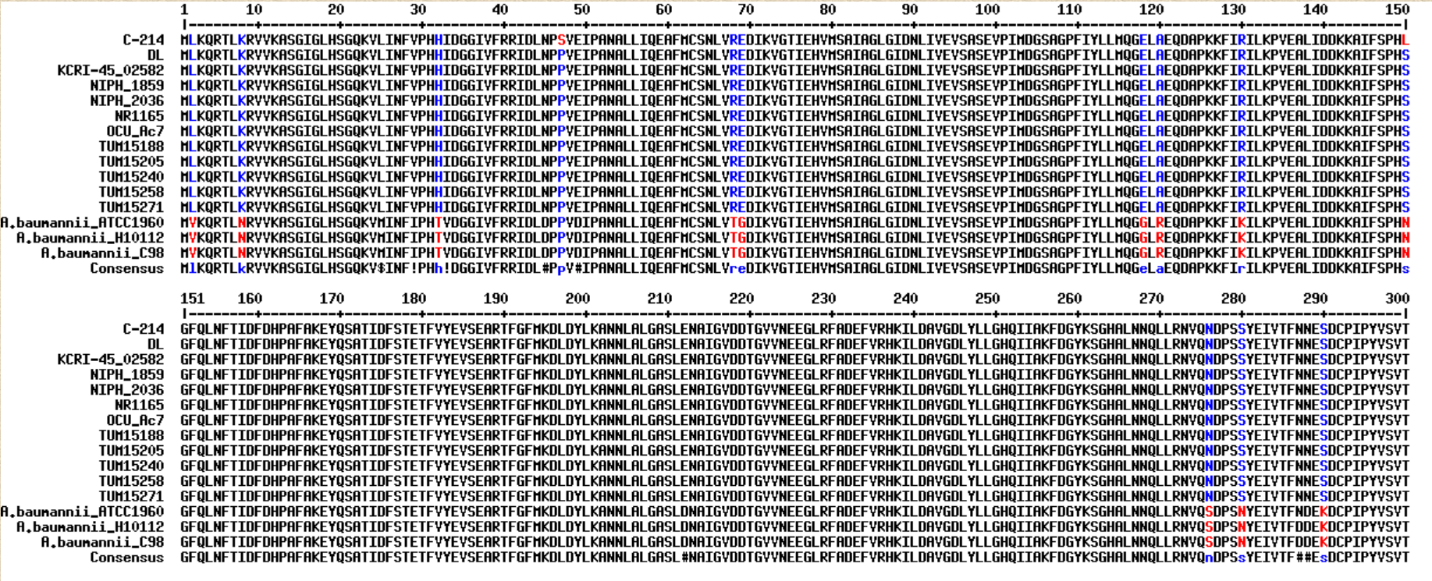
*lpxC*


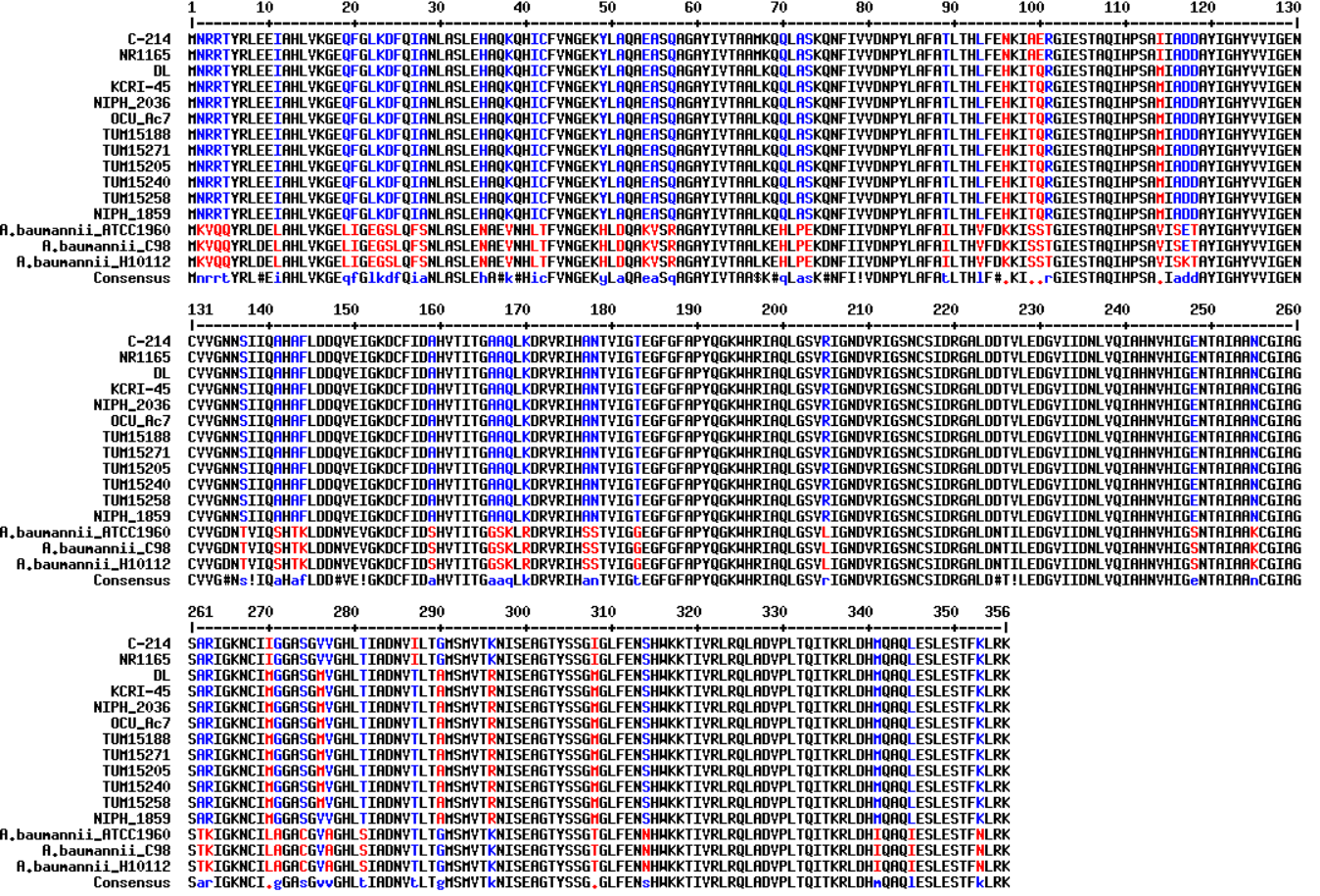
*lpxD*


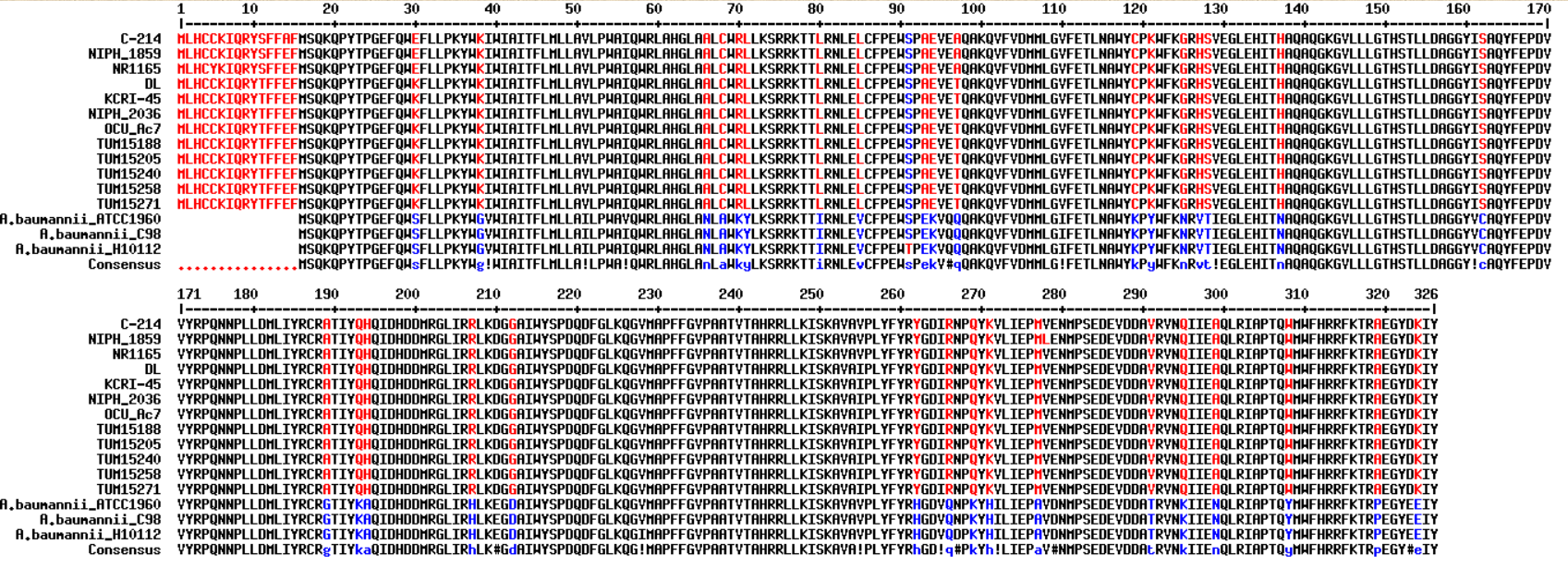
*lpxL*


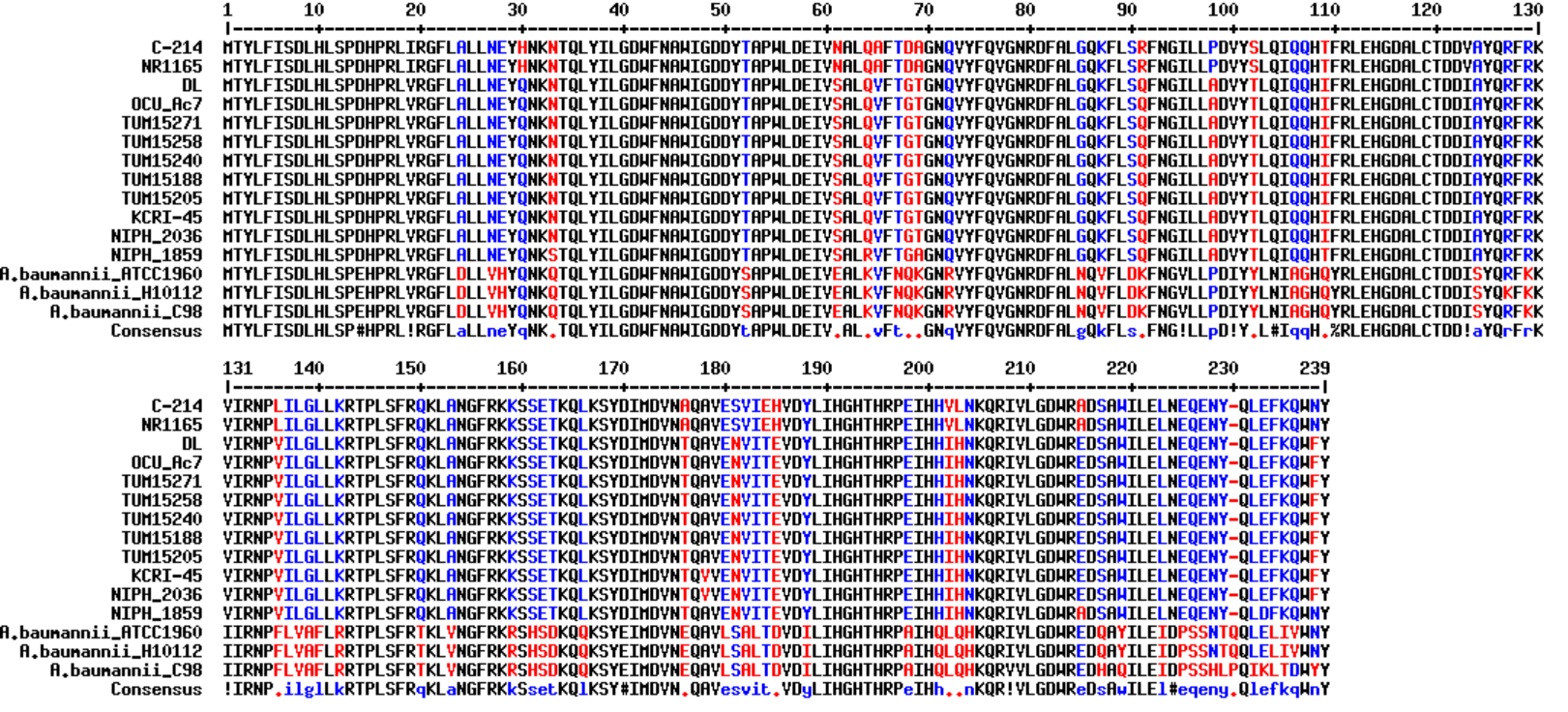
*lpxH*


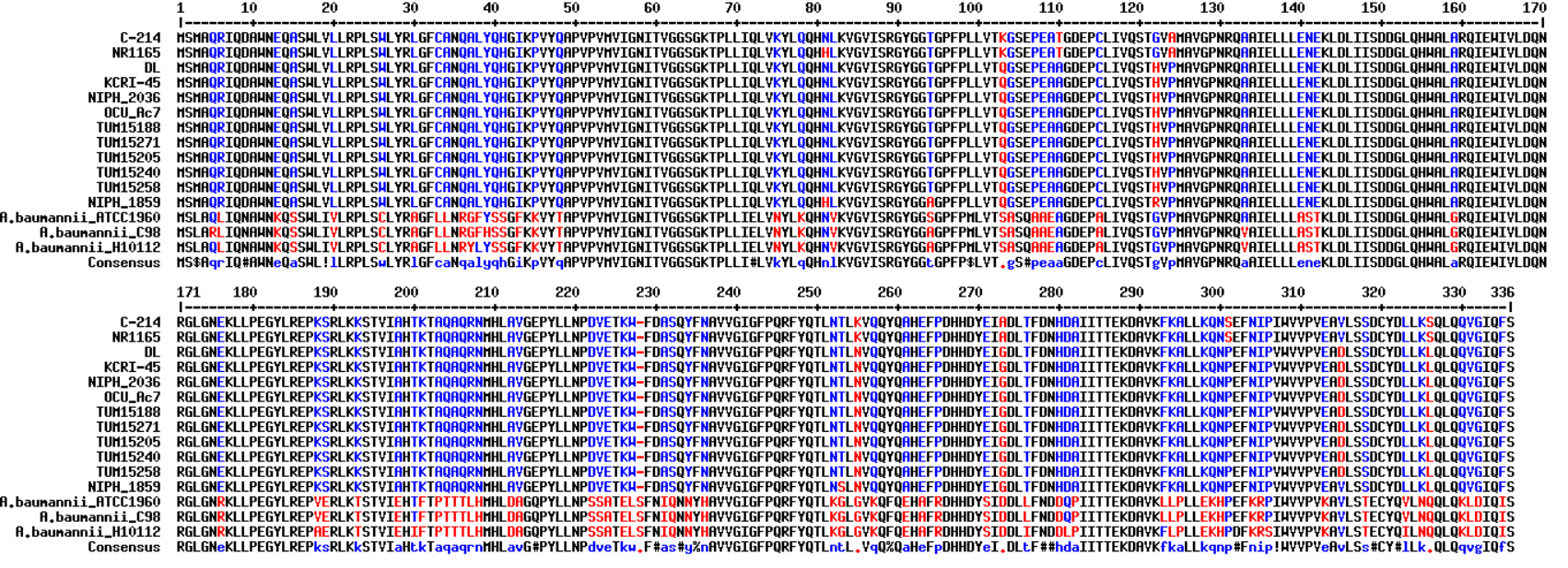
*lpxK*

**Figure S1**. Multiple sequence alignment of translated LPS gene *lpxA, lpxB, lpxC, lpxD, lpxL, lpxH* and *lpxK* of 12 *A. colistiniresistens* and three *A. baumannii* strains. Here black color sequences showing the high consensus alignment. Red color reparents low consesnsus and blue indicate neutral consensus.
